# Interaction of Glucocorticoid Receptor (GR) with Estrogen Receptor (ER) α and Activator Protein 1 (AP1) in Dexamethasone-mediated Interference of ERα Activity*

**DOI:** 10.1074/jbc.M113.473819

**Published:** 2013-07-03

**Authors:** Sudipan Karmakar, Yetao Jin, Akhilesh K. Nagaich

**Affiliations:** From the Division of Therapeutic Proteins, Office of Biotechnology Products, Office of Pharmaceutical Sciences, Center for Drug Evaluation and Research, Food and Drug Administration, Bethesda, Maryland 20892

**Keywords:** AP1 Transcription Factor, Breast Cancer, Estrogen, Estrogen Receptor, Glucocorticoid Receptor, Glucocorticoids

## Abstract

The role of glucocorticoids in the inhibition of estrogen (17-β-estradiol (E2))-regulated estrogen receptor (ER)-positive breast cancer cell proliferation is well established. We and others have seen that synthetic glucocorticoid dexamethasone (Dex) antagonizes E2-stimulated endogenous ERα target gene expression. However, how glucocorticoids negatively regulate the ERα signaling pathway is still poorly understood. ChIP studies using ERα- and glucocorticoid receptor (GR)-positive MCF-7 cells revealed that GR occupies several ERα-binding regions (EBRs) in cells treated with E2 and Dex simultaneously. Interestingly, there was little or no GR loading to these regions when cells were treated with E2 or Dex alone. The E2+Dex-dependent GR recruitment is associated with the displacement of ERα and steroid receptor coactivator-3 from the target EBRs leading to the repression of ERα-mediated transcriptional activation. The recruitment of GR to EBRs requires assistance from ERα and FOXA1 and is facilitated by AP1 binding within the EBRs. The GR binding to EBRs is mediated via direct protein-protein interaction between the GR DNA-binding domain and ERα. Limited mutational analyses indicate that arginine 488 located within the C-terminal zinc finger domain of the GR DNA-binding domain plays a critical role in stabilizing this interaction. Together, the results of this study unravel a novel mechanism involved in glucocorticoid inhibition of ERα transcriptional activity and E2-mediated cell proliferation and thus establish a foundation for future exploitation of the GR signaling pathway in the treatment of ER-positive breast cancer.

## Teaser

**Background:** How glucocorticoids affect ER-positive breast cancer cell proliferation is unclear.

**Results:** GR occupies ERα-binding regions (EBRs) via tethering to AP1 and ERα.

**Conclusion:** Interaction of GR with EBRs via ERα and AP1 inhibits E2-ERα activity.

**Significance:** Breast tumors with ERα and AP1 expression will be responsive to glucocorticoid therapy; the study establishes a foundation for personalized medicine for BC.

## Introduction

Estrogen (17-β-estradiol (E2))^2^ signaling is a key determinant of growth and survival of normal and malignant breast epithelial cells, which underscores the widespread use of anti-estrogens and aromatase inhibitors in the adjuvant treatment of breast cancer. Estrogen signaling is mainly mediated via estrogen receptor (ERα), a ligand-inducible transcription factor that regulates number of genes involved in cell proliferation, differentiation, survival, cell migration, tumor invasiveness, and normal reproductive functions. An aberrant E2 signaling or ERα gene regulatory function leads to tumor development in breast and other reproductive organs in women (1, 2). Although E2 is widely recognized for its role in breast cancer, little is known concerning a potential role of glucocorticoids (GCs) in this disease. Although numerous epidemiological and physiological studies over the past decade have indicated that chronic psychosocial stress and stress-induced GC (cortisol) contribute to the etiology of breast cancer, the validity of this assertion and the possible mechanisms involved are not well established (3–5).

GCs are key regulators of cell proliferation and are extensively used in the treatment of cancer. However, GCs cause divergent effects on the growth of different cancer cells. Although GCs inhibit cell growth and trigger apoptotic death in malignant lymphocytes, making them the mainstay of therapy for various leukemias and lymphomas, their effects on breast cancer cell proliferation is quite variable and dependent on the status of ERα expression in these cells (6). *In vitro* studies show that GCs inhibit growth of ERα-positive (MCF-7, ZR-75-1, and Con-8) cells via blocking the cell cycle at the G_0_/G_1_ phase (7–9). By contrast, ERα-negative (MDA-MB-231) cells show no growth inhibition by GCs, indicating that GCs block breast cancer cell proliferation by obstructing the ERα signaling pathway. Instead, recent studies have shown that GCs initiate a survival signal in ERα-negative breast epithelial (MCF10A) and cancer (MDA-MB-231) cells via up-regulation of pro-survival genes, such as serum and glucocorticoid-regulated kinase 1 (SGK1) and dual specificity phosphatase 1 (DUSP1) (10, 11). Additionally, numerous studies have indicated that GCs inhibit apoptosis of both ER-positive and ER-negative breast cancer cells induced by agents such as doxorubicin (12), trastuzamab (13), and paclitaxel (14). Moreover, breast cancer xenograft study in mice has shown that pretreatment with synthetic GC dexamethasone (Dex) decreases tumor response to paclitaxel chemotherapy by inhibition of tumor cell apoptosis (15, 16). Although several of these studies indicate that GCs play an important role in E2 signaling in breast cancer, the molecular mechanisms underlying such effects and the intracellular pathways involved are not understood.

Cellular actions of GCs are mediated by binding to its cognate intracellular receptor, GR. Both GR and ER are ligand-activated transcription factors (TFs) belonging to the nuclear receptor (NR) superfamily (17). These receptors primarily reside in the cytoplasm where they remain associated with heat shock proteins. Upon ligand binding, both receptors shed the heat shock proteins, translocate to the nucleus, dimerize, and get recruited to the regulatory regions of their target genes either by directly binding to specific hormone response elements or by indirectly tethering through transcription factors such as AP1 (18–21), Sp1 (22–24), signal transducer and activator of transcription (Stat1) (25), and NFκB (26–28). This is followed by the recruitment of various coregulators such as steroid receptor coactivators (SRCs), pioneer factor (FOXA1), histone acetyltransferases (cyclic AMP-binding protein, CBP and E1A-binding protein, p300), histone methyltransferases (coactivator-associated arginine methyltransferase 1, CARM1 and protein arginine methyltransferase 1, PRMT1), and ATP-dependent chromatin remodeling complex (human SWItch/Sucrose NonFermentable, hSWI/SNF) that remodel the chromatin structure and make the DNA accessible to RNA polymerase II and other basal transcriptional machinery, leading to activation or repression of gene transcription (17, 29). Although there are a number of studies implicating the GR and ERα cross-talk with other “partnering” TFs in their transrepression function, how GR antagonizes ERα function is not clear.

To understand how activated GR counteracts the ERα signaling pathway and regulates survival of ERα-positive breast cancer cells, we carried out cell proliferation, gene expression, and ChIP assays in ERα- and GR-positive MCF-7 breast cancer cells, in the presence of E2 or Dex, alone or in combination. We found that in the presence of E2, Dex significantly inhibits E2-dependent proliferation of MCF-7 cells and down-regulates expression of key ERα target genes (*pS2* and *Cyclin D1*). ChIP assays showed that GR is recruited to ERα-binding regions (EBRs) in the presence of Dex and E2, leading to the destabilization of the ERα transcriptional complex. Binding of GR to EBRs was found to be regulated by the recruitment of pioneer factor FOXA1 and AP1. Protein-protein interaction studies showed that GR directly interacts with ERα through its DNA-binding domain (DBD). Mutational studies indicated that GR-DBD plays a critical role in stabilizing GR-ERα interactions. Together, results of this study show that a direct interaction between GR and ERα, mediated via AP1, plays an important role in the regulation of ERα activity and GR-mediated growth inhibition of E2-induced ERα-positive breast cancer cells.

## EXPERIMENTAL PROCEDURES

### 

#### 

##### Materials

E2, Dex, doxycycline (DOX), and ICI 182,780 (an estrogen receptor antagonist) were obtained from Sigma. The vehicle (VEH) for all the experiments was 0.1% ethanol. The siRNA targeting FOXA1 (5′-GAGAGAAAAAAUCAACAGC), described previously (30) and prevalidated as silencer® select siRNA, was obtained from Invitrogen. Ambion® silencer® select negative control #2 (Invitrogen) was used as a nonspecific siRNA control. Sp1 (ON-Targetplus SMRTpool) and control siRNA (ON-Targetplus Control pool) were purchased from Dharmacon (Thermo Scientific, Waltham, MA). Human GR, produced in baculovirus, was purchased from Invitrogen. Plasmids pCR3.1-ERα encoding full-length human ERα and pERE-E1b-Luc containing E2-responsive reporter gene have been described previously (31, 32). The plasmid pSG5-GR encoding full-length rat GR was kindly provided by Dr. Michael Garabedian (New York University Langone Medical Center School of Medicine). The DBD point mutants R466K and R488Q of rat GR were generous gifts from Dr. Keith Yamamoto (University of California San Francisco) (33). GST-fused ERα encoding amino acid (aa) residues 1–250 (GST-ERα-N), aa 251–595 (GST-ERα-C), and GST-GR (N-terminal: aa 106–318, DBD: aa 435–510, and LBD: aa 589–771) and DBD (R488Q) were produced in *Escherichia coli* and affinity-purified on glutathione beads.

##### Cell Culture and Growth Assays

MCF-7 and MDA-MB-468 human breast cancer cells and HeLa human cervical carcinoma cells were obtained from American Type Cell Culture (Manassas, VA) and maintained in DMEM supplemented with 10% FBS. MCF-7 Tet-Off TAM-67 cell line was a generous gift from Dr. Powel H. Brown (M. D. Anderson Cancer Center, Houston, TX). This cell line was maintained in DMEM supplemented with 10% FBS, 100 μg/ml hygromycin, and 1 μg/ml DOX. For growth assays, MCF-7 cells (1 × 10^5^) were seeded in each well of a 6-well plate and grown overnight in DMEM containing 10% FBS. The next day, cells were washed with PBS and fed with DMEM containing 10% hormone-depleted, charcoal-stripped FBS (sFBS) (day 0). Next, the cells were treated with VEH, E2 (1 nm), Dex (100 nm), or E2 (1 nm) + Dex (100 nm) and allowed to grow for 5 days with medium plus hormone replacement on day 3. The cells were harvested on days 5 with 0.05% trypsin-EDTA (Invitrogen) and stained with trypan blue (Sigma), and the viable cell number was determined with a hemocytometer.

##### Reverse Transcription-Quantitative Polymerase Chain Reaction (RT-qPCR)

**FIGURE 1.**
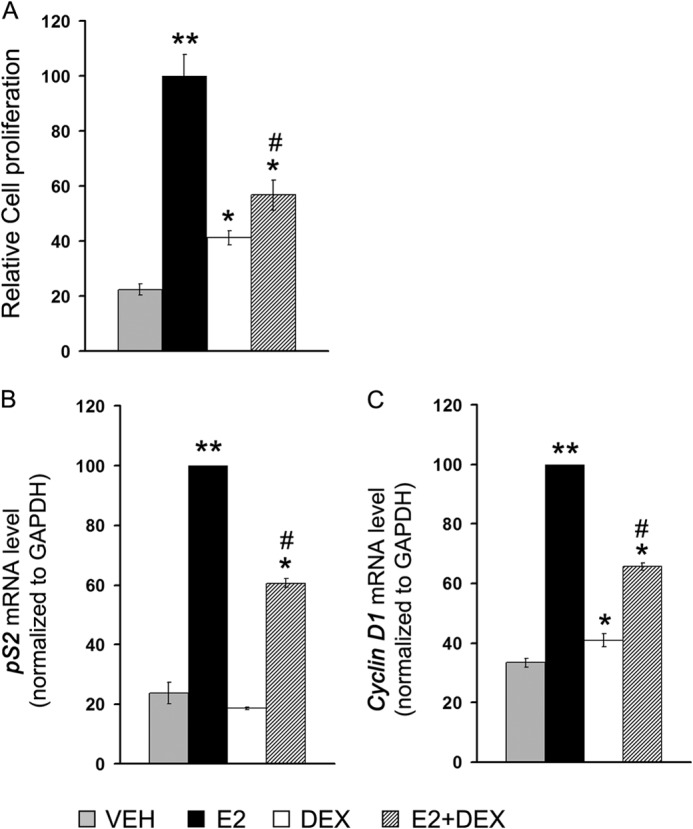
**Effect of hormones on MCF-7 cell proliferation and endogenous ERα target gene expression.** *A*, MCF-7 cells cultured in phenol red-free DMEM containing 10% sFBS were treated with VEH, E2 (1 nm), Dex (100 nm), or E2+Dex (1 nm + 100 nm) and allowed to grow for 5 days. The cells were harvested, stained with trypan blue, and counted by hemocytometer. All the treatments were performed in triplicate, and the experiment was repeated three times. The numbers of trypan blue excluding viable cells were plotted as relative cell proliferation, setting the number corresponding to E2-treated cells as 100. *B* and *C*, MCF-7 cells were grown for 48 h under similar condition as above prior to hormone treatment (3 h for *Cyclin D1* and 24 h for *pS2*). Next, cells were harvested for RNA isolation and mRNA levels specific to *Cyclin D1* and *pS2* evaluated by RT-qPCR. In each case, data were normalized to corresponding GAPDH RNA values. The experiments were repeated three times in triplicate, and the data were plotted as relative mRNA level, setting the number corresponding to E2-treated cells as 100. A *single asterisk* (*) denotes statistical significance at *p* < 0.05, and *double asterisks* (**) denote statistical significance at *p* < 0.001 compared with vehicle control. The number sign (#) denotes significance at *p* < 0.05 *versus* E2-treated group.

To determine the expression of endogenous ERα target genes, MCF-7 cells were grown in DMEM with 10% FBS. After 24 h, the cells were washed with PBS and then switched to phenol red-free DMEM with 10% sFBS. After 48 h, the cells were treated with VEH, E2 (1 nm), Dex (100 nm), or E2 (1 nm) + Dex (100 nm) for 3 h (for *Cyclin D1*) or 24 h (for *pS2*) and harvested for RNA isolation using a Qiagen kit. Duration of hormone treatment was chosen based on previous studies that showed maximum E2-induced mRNA expression of *pS2* and *Cyclin D1* genes at these time points in MCF-7 cells (34, 35). RNA was reverse transcribed using a Bio-Rad iScript cDNA synthesis kit. The reverse transcribed DNA was quantified by qPCR using SYBR® Green-based detection (see Fig. 1). Primer sequences for *pS2* (36) and *Cyclin D1* (37) genes have been described. The mRNA levels of *GAPDH* (used as an internal reference gene) were quantified using the following primer sequences: forward, 5′-GAGTCAACGGATTTGGTCGT-3′; and reverse, 5′-GACAAGCTTCCCGTTCTCAG-3′.

##### Chromatin Immunoprecipitation Assays

**FIGURE 2.**
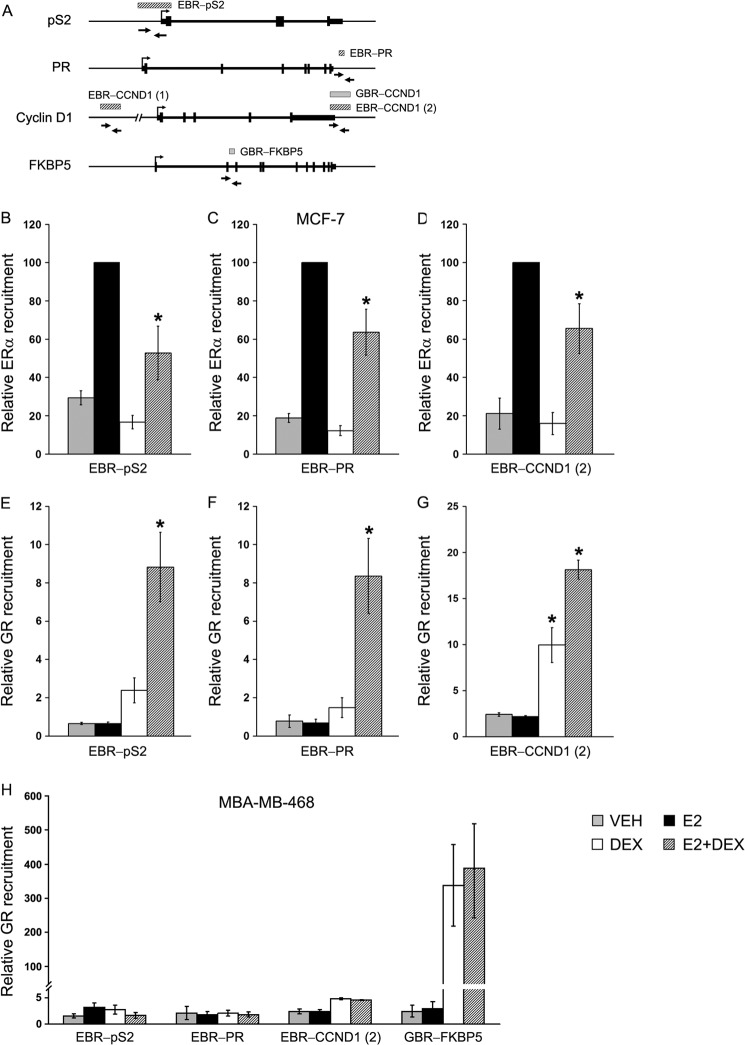
**Ligand-activated GR interacts with EBRs located in the promoter/enhancer regions of *pS2*, *Cyclin D1*, and *PR* genes.** *A*, a schematic diagram showing open reading frames of *pS2*, *PR*, and *Cyclin D1* genes and the locations of ERα-binding (*striped horizontal bar*) and GR-binding (*gray horizontal bar*) regions determined by previous ERα ChIP-chip (40) and GR ChIP-seq studies (43). *B–G*, chromatin prepared from VEH, E2 (10 nm), Dex (100 nm), or E2-(10 nm) + Dex-treated (100 nm) MCF-7 cells was subjected to ChIP assay using antibodies for ERα (*B–D*) or GR (*E–G*). Immunoprecipitated DNA was quantified by qPCR using primers for EBR-pS2 (*B* and *E*), EBR-PR (*C* and *F*), and EBR-CCND1-(2) (*D* and *G*); locations of primers are shown in the schematic diagram (*A*), and the sequences are in Table 1. The experiment was repeated three times, and the data are plotted as relative factor recruitment (average ± S.E.), setting the number corresponding to ERα recruitment in E2-treated cells as 100. The *asterisk* (*) denotes statistical significance at *p* < 0.05 in comparison with E2-treated group for *B–D* and in comparison with VEH control for *E–G. H*, An ERα-negative and GR-positive cell line MDA-MB-468 was treated with VEH, E2 (10 nm), Dex (100 nm), or E2 (10 nm) + Dex (100 nm) for 45 min and subjected to ChIP assay with anti-GR antibodies. ChIPed DNA was amplified with primers specific for EBR-pS2, EBR-PR, EBR-CCND1-(2), and GBR-FKBP5.

ChIP assays were performed as described earlier with minor modifications (37). MCF-7 cells were grown for at least 48 h in phenol red-free DMEM supplemented with sFBS prior to hormone treatment. Thereafter, cells were treated with VEH, E2 (10 nm), Dex (100 nm), or E2 (10 nm) + Dex (100 nm) for 45 min and cross-linked for 10 min with formaldehyde (1% v/v). For ChIP assays with siRNA-transfected cells, MCF-7 cells were transfected either with 50 nm siRNA (control) or siRNA (FOXA1) using Oligofectamine transfection reagent (Invitrogen). Identical siRNA transfection strategy was applied for depletion of Sp1 in MCF-7 cells, except that 20 nm SMRT pool of siRNA was used instead of 50 nm. After 4–6 h of transfection, the medium was switched to phenol red-free DMEM supplemented with sFBS. After 72 h, the cells were treated with hormones for 45 min as described before prior to the ChIP assay. Similarly, MCF-7 Tet-Off TAM-67 cells were grown for 24 h, washed with PBS, switched to phenol red-free DMEM containing sFBS plus or minus DOX, and cultured for 3 days prior to the hormone treatment and ChIP assay. The cells were harvested, lysed, and sonicated, and the lysate was precleared by protein A/G-agarose (Millipore, Billerica, MA). The precleared chromatin was immunoprecipitated with antibodies against ERα (HC-20 and H-184), GR (H-300 & M-20), SRC-3 (C-20), or normal rabbit/goat IgG (all antibodies were obtained from Santa Cruz Biotechnology, Santa Cruz, CA). The immunoprecipitated chromatin was collected on protein A/G-agarose beads preblocked with salmon sperm DNA. The cross-linking was reversed by heating the beads at 65 °C overnight. The purified immunoprecipitated DNA was quantified by qPCR using SYBR® green chemistry and normalized against input chromatin. Locations of primers used to amplify ChIPed DNA are shown in Fig. 2*A*, and the primer sequences are shown in Table 1.

**TABLE 1:** **Primer sequences used for ChIP assay**

Sequence tags/genes	Primer sequences
EBR-pS2	
Forward	5′-CTCCCGCCAGGGTAAATA-3′
Reverse	5′-GGCCAAGCCTTTTTCC-3′
EBR-PR	
Forward	5′-AATGAGGCTGACATTCTGGGA-3′
Reverse	5′-GTTGACCTCATTCCAAGGCAG-3′
EBR-CCND1 (1)	
Forward	5′-GCTCTTTACGCTCGCTAACC-3′
Reverse	5′-GGGCAGATCTCGACTAGGAA-3′
EBR-CCND1 (2)	
Forward	5′-CAGTTTGTCTTCCCGGGTTA-3′
Reverse	5′-TCATCCAGAGCAAACAGCAG-3′
GBR-FKBP5	
Forward	5′-CCACATCAAGCGAGCTGCAAAAA-3′
Reverse	5′-GCCAGCCACATTCAGAACAGGGT-3′

##### Coimmunoprecipitation Assay and Western Blotting

For the coimmunoprecipitation assay, MCF-7 cells were grown in phenol red-free DMEM supplemented with 10% sFBS for at least 24 h prior to hormone treatment. Subsequently, the cells were treated with VEH, E2 (10 nm), Dex (100 nm), or E2 (10 nm) + Dex (100 nm) for 1 h and harvested in PBS containing protease inhibitors (Roche Applied Science). Cell lysates were prepared by incubating cell pellets in the immunoprecipitation lysis buffer (50 mm Tris-HCl, pH 7.5, 5 mm EDTA, 150 mm NaCl, 0.5% Nonidet P-40, 5% glycerol) supplemented with protease and phosphatase inhibitors (Halt protease and phosphatase inhibitor mixture; Thermo Scientific) for 20 min at 4 °C, followed by centrifugation at 14,000 × *g* for 15 min at 4 °C. Cell lysates were precleared for 1 h with protein A/G-agarose (Santa Cruz Biotechnology) beads and incubated overnight with anti-ERα (HC-20) antibody at 4 °C. The immune complex was collected on protein A/G-agarose beads, washed three times with immunoprecipitation lysis buffer, eluted with sample buffer (50 mm Tris-HCl, pH 6.8, 2% SDS, 10% glycerol, 1% β-mercaptoethanol, 12.5 mm EDTA, 0.02% bromphenol blue), resolved by 10% SDS-PAGE, transferred to PVDF membranes (Millipore), and probed first with primary antibodies followed by alkaline phosphatase-conjugated affinity-purified anti-mouse or anti-rabbit IgGs and developed using chemiluminescent substrate (Tropix CSPD; Invitrogen). The Western blot signals were imaged on XO-1 blue film (Kodak). Following primary antibodies were used both for Western blotting and immunoprecipitation assays: anti-ERα (HC-20), anti-GR (H-300), anti-FOXA1 (2F83; Abcam), anti-Cyclin D1 (554180; BD Pharmingen), anti-PR (1294; a kind gift from Prof. Dean Edwards, Baylor College of Medicine, Houston, TX), and anti-actin (MAB1501R; Millipore).

##### GST Pulldown Assay

All GST fusion constructs were made in pGEX-6P1 expression plasmid (GE Healthcare), and recombinant proteins were overexpressed in *E. coli* BL-21 (DE3) and affinity-purified using glutathione beads. For *in vitro* GR and ERα interaction studies, glutathione beads coupled to ERα-GST protein were incubated with baculovirus-expressed, partially purified human GR protein in mammalian cell lysis buffer (50 mm Tris, pH 8.0, 5 mm EDTA, 150 mm NaCl, 0.5% Nonidet P-40) containing protease inhibitors for 1 h at 4 °C. Unbound proteins were removed by two washes with the lysis buffer and two washes with the lysis buffer containing 500 mm NaCl followed by a wash with PBS. The bound proteins were resolved by SDS-PAGE, transferred onto PVDF membrane (Millipore), and analyzed for GR by Western blotting. For the reverse pulldown assay, ERα was *in vitro* translated using the TnT system (Promega, Madison, WI) and incubated with GST-GR proteins immobilized to glutathione beads. The protein-bound beads were washed as described before and probed for ERα using anti-ERα antibody (HC-20; Santa Cruz Biotechnology).

##### Transactivation Assays

For ERα transactivation assays, HeLa cells (2 × 10^5^) were seeded in each well of 6-well plate in DMEM supplemented with 10% FBS. Plasmid DNAs were transfected using Lipofectamine and OptiMEM (Invitrogen). After 4–6 h of transfection, the cells were switched to phenol red-free DMEM containing 10% sFBS. After 48 h, the cells were treated with VEH or E2 (1 nm) for additional 24 h. Next, the cells were harvested, lysed, and assayed for luciferase activity using luciferase detection kit (Promega). Luciferase signals were acquired using Glomax 96-Microplate Luminometer (Promega). Values were normalized to total protein content measured using BCA protein assay kit (Pierce).

## RESULTS

### 

#### 

##### Ligand-activated GR Inhibits MCF-7 Cell Proliferation and Represses ERα Transcriptional Activity

To determine the mechanism underlying GR-mediated repression of ERα activity and the subsequent effect on E2-ERα-mediated cell proliferation, we used an ERα- and GR-positive human breast adenocarcinoma cell line MCF-7 as a model system. To test the responsiveness of this cell line to ERα and GR ligands, cell proliferation and ERα transcriptional activity studies were conducted in presence the of physiological concentration of E2 (1 nm) and Dex (100 nm), alone or in combination. E2 treatment showed a 5-fold increase in MCF-7 cell proliferation, whereas Dex alone exhibited less than 2-fold change (Fig. 1*A*). However, when cells were treated with E2 and Dex in combination, a repressive effect on E2-mediated cell proliferation was observed. This observation indicated a clear role of ligand-activated GR in inhibiting E2-ERα-dependent MCF-7 cell proliferation. A higher concentration (1–10 μm) of Dex showed only a marginal increase in the inhibition of E2-mediated cell proliferation than 100 nm Dex, suggesting that partial inhibition of E2-induced cell proliferation is not due to subsaturated hormone-bound GRs in the cells (data not shown). To further assess the effect of Dex on ERα activity, the expression levels of endogenous ERα target genes *pS2* and *Cyclin D1* were measured in the presence of E2 and Dex, alone or in combination. Consistent with the growth assay, E2 treatment led to a significant increase in both *pS2* and *Cyclin D1* gene expression (Fig. 1, *B* and *C*). Dex alone did not show any effect on expression of either of these genes. However, with E2+Dex, the expression of both genes was repressed compared with that observed in the presence of E2 only. Decreased mRNA expression of *Cyclin D1* and *PR* genes was correlated with decreased *Cyclin D1* and *PR* proteins in presence of E2+Dex than E2-treated cells, confirming that E2+Dex down-regulates expression of these genes (data not shown). Together, the results of these experiments indicated that Dex inhibits E2-induced ERα activity and E2-induced proliferation of MCF-7 cells.

To gain insight into the role of GR on ERα transcriptional activity, the interaction of GR with EBRs was investigated using MCF-7 cells. Three genes: *pS2*, *PR*, and *Cyclin D1*, were chosen for this study because we and others have shown that expression of these genes in MCF-7 cells is repressed by E2+Dex, compared with E2 alone (38, 39). These genes harbor functional EBRs in the neighborhood that were identified in a recent genome-wide ChIP-chip study (40). Furthermore, a chromatin interaction network study of ERα in MCF-7 cells showed that these EBRs are functionally involved in the regulation of their respective target genes (41). In a recent study, we confirmed E2-dependent recruitment of ERα to these binding sites (42). These sites include EBR-pS2 at 300 bp upstream of the *pS2* transcriptional start site, EBR-PR located at ∼5 kb downstream of the PR 3′-untranslated region, and EBR-CCND1-(2) at 500 bp downstream of *Cyclin D1* coding region (Fig. 2*A* and Table 2). Of these EBRs, only EBR-CCND1-(2) has an overlapping GR-binding region as observed in a GR ChIP-Seq study performed in a human lung cancer cell line (A549) treated with Dex (herein referred as GBR-CCND1) (43). Of note, EBR-CCND1-(2) does not have consensus ERE or GRE sequence, suggesting that ERα or GR binding to this region is indirect.

**TABLE 2:** **Chromosomal positions and locations (with respect to the start of the sequence tags) of transcription factor binding sites within EBRs and GBR** Publicly available bed files from the genome-wide ChIP-chip analysis for ERα in MCF-7 cells (40) or ChIP-seq analysis for GR in A549 cells (43) were uploaded in the UCSC genome browser. DNA sequences corresponding to ERα- and GR-binding regions around *pS2*, *PR*, and *Cyclin D1* genes were retrieved from the UCSC database. Locations of putative binding sites for ERα, GR, AP1, and Sp1 in these regions were predicted using the Genomatix MatInspector program (73).

Sequence tags	Chromosomal Position of EBR/GBR	Size	Location and sequences of TF binding sites within EBRs and GBR
		*bp*	
EBR-pS2 (ER_10218)	Chr21:42659377–42660167	791	AP1 (669–676) 5′-TGATTCA-3′
			ERα (730–743) 5′-GGCCACCGTGACC-3′
			Sp1 (749–758) 5′-GGGGAAGGG-3′
EBR-PR (ER_7204)	Chr11:100409715–100410667	953	AP1 (319–326) 5′-TGATTCA-3′
			ERα (438–451) 5′-GGTCAGCATGACA-3′
			Sp1 (563–575) 5′-GGGCGCAGCCCC-3′
EBR-CCND1 (1)	Chr11:69162761–69163409	649	Sp1 (114–124) 5′-GGGGCTGGGC-3′
(ER_7070)			(306–317) 5′-GCCGCGCCCC-3′
EBR-CCND1 (2)	Chr11:69177826–69179657	1832	AP1 (637–642) 5′-TGAGCC-3′
(ER_7072)			(1155–1162) 5′-TGAATCA-3′
			(1816–1823) 5′-TGGCTCA-3′
			Sp1 (244–254) 5′-ACCCCGCCCC-3′
			(349–359) 5′-TGATGGGGCA-3′
GBR-FKBP5	Chr6:35677579–35678104	526	AP1 (62–69) 5′-TGACTTA-3′
(GR_3100)			(209–216) 5′-TGACTTA-3′
			GR (259–274) 5′-AGAACACCCTGTTCT-3′

To elucidate the mechanism involved in GR-mediated repression of ERα activity, we tested the possibility of recruitment of GR to these EBRs in response to E2+Dex treatment. As expected, E2 treatment led to strong induction of ERα interaction with the EBRs (Fig. 2, *B–D*). Dex treatment alone did not show any effect on ERα recruitment compared with vehicle control. Interestingly, the combination of Dex and E2 inhibited ERα recruitment to all the three EBRs compared with E2 treatment alone. We noted that E2+Dex treatment caused partial inhibition of ERα recruitment to EBRs, whereas ICI 182,780 caused complete inhibition, indicating perhaps a less potent inhibitory mechanism employed by Dex-induced GR (data not shown). By contrast, recruitment of GR to EBRs showed a different profile. As expected, there was no loading of GR to EBRs in the presence of E2 (Fig. 2, *E–G*). GR was also minimally recruited to EBRs in the presence of Dex, except to the EBR-CCND1-(2) that harbors a GBR overlapping the EBR in this region (Fig. 2*A*) (43). Surprisingly, when cells were treated with E2+Dex, there was a synergistic enrichment of GR to each of the EBRs (Fig. 2, *E–G*). This enrichment is highly specific because no such recruitment of GR is observed at unrelated site or with IgG control (data not shown). These results indicated that there may be a correlation between increase of GR and decrease of ERα recruitment in response to E2+Dex treatment leading to GR-mediated repression of these genes.

To test whether ERα causes E2+Dex-mediated recruitment of GR to EBRs, we conducted a ChIP assay with the ERα-negative and GR-positive breast cancer cell line MDA-MB-468. Cells were treated with hormones as before, and chromatin was immunoprecipitated with anti-GR antibodies. We observed no notable recruitment of GR to either of the EBRs tested in the presence of E2+Dex (Fig. 2*H*). To ensure that GR is responsive to Dex and functionally active in this cell line, we measured the recruitment of GR to a known GR-binding site located in the regulatory region of GR target gene FKBP5. This site is located in intron 2 of the FKBP5 gene and contains a canonical GRE (AGAACAgggTGTTCT) (Fig. 2*A* and Table 2). Recruitment of GR to this site (referred as GBR-FKBP5 in this study) in response to Dex treatment was shown in A549 human lung cancer cell line by both regular ChIP and ChIP-seq experiments (43, 44). A qPCR of the DNA isolated by ChIP using anti-GR antibody showed a significant loading of GR to this site, indicating that the lower level of GR recruitment to EBRs in MDA-MB-468 cells is not due to impaired DNA binding of GR in this cell line (Fig. 2*H*).

##### GR Recruitment to EBRs Destabilizes the ERα-SRC-3 Complex, Leading to Inhibition of ERα-mediated Transcription

To determine whether GR binding to EBRs affects the ERα-mediated transcriptional complex, we tested the recruitment of ERα coregulatory protein, SRC-3 to EBRs by ChIP assay. SRC-3 is a member of p160 family of steroid receptor coactivators that plays a critical role in ERα-mediated transcriptional program. The SRC-3 protein harbors a steroid receptor recognition domain-containing L*XX*LL motif and coordinates the recruitment of histone-modifying and chromatin-remodeling proteins through its CBP/p300 interaction domain and a histone acetyl transferase domain (45). Several studies have shown that depletion of SRC-3 in MCF-7 cells critically impairs ERα-mediated transcription and inhibits E2-induced growth of MCF-7 cells (46). Furthermore, the SRC-3 gene is frequently amplified in breast cancer, and increased SRC-3 levels have been correlated with poor clinical outcome in breast cancer patients (47–49).

**FIGURE 3.**
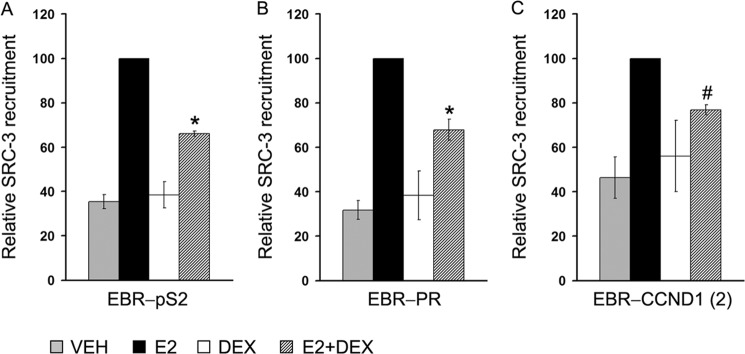
**Loss of SRC-3 recruitment to EBRs in response to E2+Dex treatment.** MCF-7 cells treated with VEH, E2 (10 nm), Dex (100 nm), or E2 (10 nm) + Dex (100 nm) were subjected to ChIP assay with anti-SRC-3 antibody followed by qPCR detection of immunoprecipitated DNA with primers specific for EBR-pS2 (*A*), EBR-PR (*B*), and EBR-CCND1-(2) (*C*). The data represent the averages ± S.E. of three independent experiments. In each case, recruitment of SRC-3 to EBRs in E2-treated cells was set as 100. The *asterisk* (*) denotes a *p* value of <0.05, and # denotes a *p* value of 0.071 *versus* E2-treated group.

To test how E2+Dex treatment affects SRC-3 recruitment to these EBRs, we conducted a ChIP assay with hormone-deprived MCF-7 cells. As expected, E2 treatment led to a robust recruitment of SRC-3 to all the three EBRs tested, whereas Dex alone did not show any effect. By contrast, E2+Dex treatment significantly decreased SRC-3 loading to all the EBRs compared with E2 treatment alone (Fig. 3). Together, our data indicate that Dex-induced GR recruitment to EBRs not only impairs ERα binding but also destabilizes the ERα-SRC3 complex, leading to inhibition of transcription.

##### FOXA1 Regulates GR Recruitment to EBRs via ERα

**FIGURE 4.**
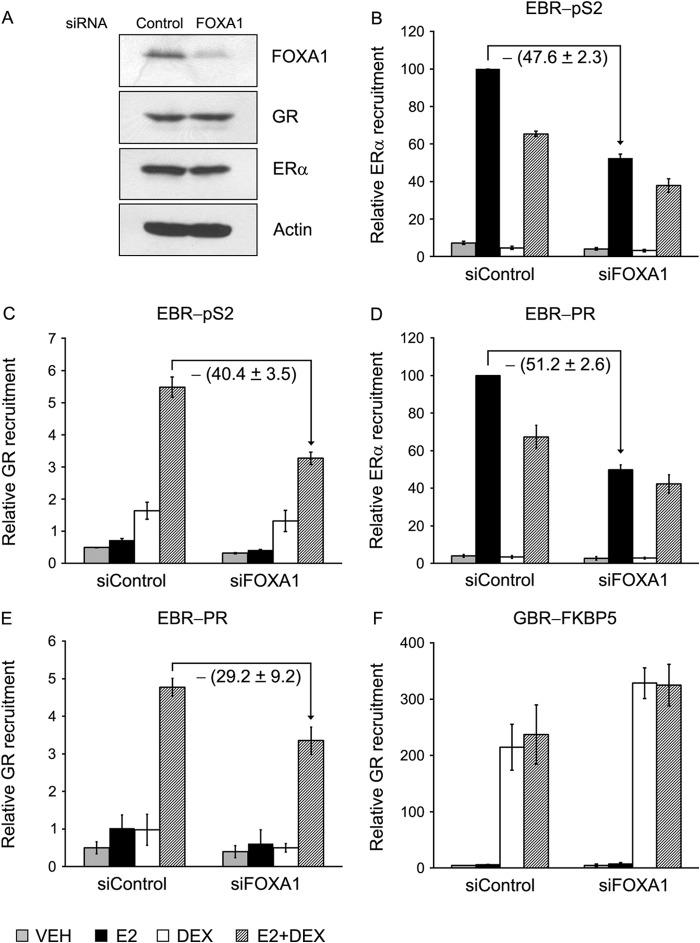
**FOXA1 depletion by siRNA decreases recruitment of ERα and GR to EBRs.** *A*, MCF-7 cells were transfected with control or FOXA1 siRNA. After 72 h, the cells were harvested and lysed, and the lysate was analyzed for ERα, GR, FOXA1, and actin proteins by Western blotting. *B–F*, a parallel set of siRNA transfected cells was treated with VEH, E2 (10 nm), Dex (100 nm), or E2 (10 nm) + Dex (100 nm) for 45 min as before and subjected to ChIP assay using anti-ERα (*B* and *D*) and anti-GR (*C–E*) antibodies. Immunoprecipitated DNA was quantified by qPCR using primers for the EBR-pS2 (*B* and *C*), EBR-PR (*D* and *E*), or GBR-FKBP5 (*F*). The data represent the average relative recruitment of three independent experiments; the *error bars* represent standard error of the mean (S.E.).

To assess the role of ERα in GR recruitment to EBRs, an indirect approach was taken. Multiple genome-wide ERα interaction studies have shown that the forkhead box protein FOXA1 acts as a pioneer factor and a coactivator for ERα-mediated transcriptional response (30, 50). A knockdown of FOXA1 in MCF-7 cells leads to substantial decrease of ERα binding to target sites in chromatin (51), suggesting that FOXA1 is a major determinant of E2-ERα activity. To test whether decreased ERα recruitment caused by FOXA1 depletion has any effect on GR recruitment to EBRs, MCF-7 cells were transfected with siRNA control or siRNA-FOXA1. Transfection of siRNA-FOXA1 led to selective depletion of over 80–90% endogenous FOXA1 in MCF-7 cells without affecting the expression levels of ERα and GR (Fig. 4*A*). ChIP assay showed that depletion of FOXA1 decreased the loading of ERα (Fig. 4, *B* and *D*, *black bars*) to EBRs by ∼50%. Concomitantly, the recruitment of GR to EBRs decreased to a similar extent (Fig. 4, *C* and *E*, *striped bars*), indicating a correlation in the binding activity of the two receptors at the ERα-binding sites. The recruitment of GR to GBR-FKBP5, however, remained unaffected by the depletion of FOXA1, suggesting that loading of GR to GBR did not require the assistance of FOXA1 (Fig. 4*F*). These results demonstrate that increased levels of ERα recruitment to EBRs leads to increased E2+Dex-mediated GR binding to EBRs. We suggest that FOXA1 pioneering activity increases chromatin accessibility and facilitates ERα-chromatin interactions at EBRs. This increased chromatin accessibility may also affect GR binding to EBRs.

##### GR Recruitment to EBRs Is Dependent on AP1 Binding

**FIGURE 5.**
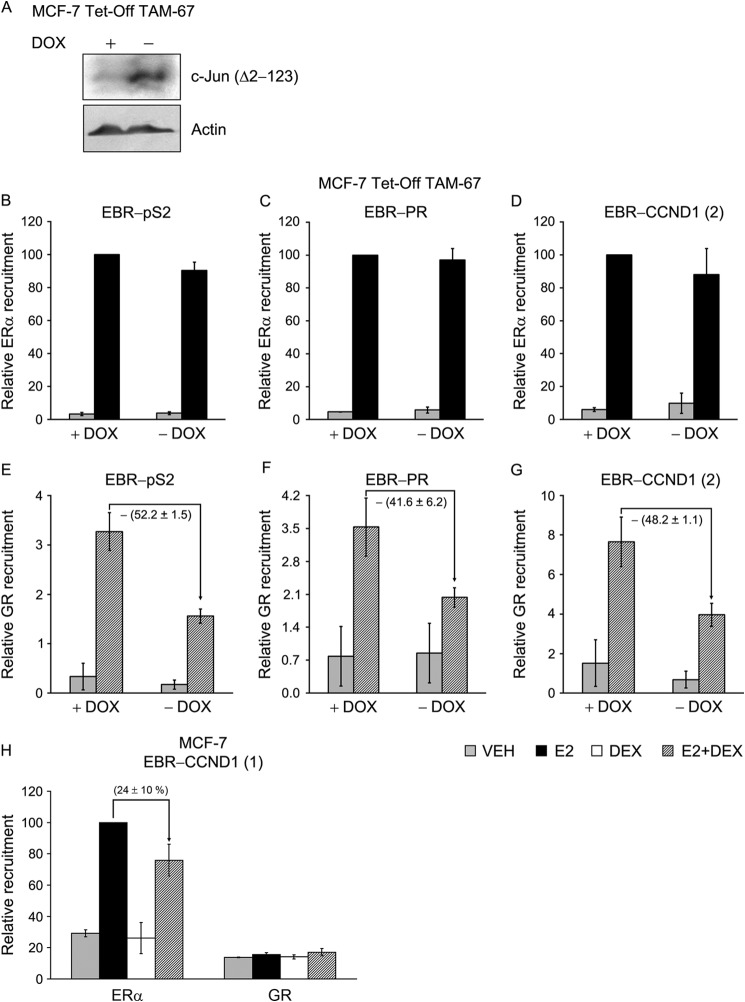
**Expression of dominant negative AP1 impairs GR recruitment to EBRs.** An engineered MCF-7 cell line expressing tet-regulated FLAG-tagged TAM-67 (c-Jun Δ2–123) was grown in the presence or absence of DOX for 3 days. *A*, expression of TAM-67 in cells grown in ± DOX was tested by Western blotting using anti-FLAG antibody; withdrawal of DOX leads to a robust expression of TAM-67. *B–G*, a parallel set of cells was treated with VEH, E2 (10 nm), or E2 (10 nm) + Dex (100 nm) for 45 min and subjected to ChIP assay with anti-ERα (*B–D*) and anti-GR (*E–G*) antibodies. ChIPed DNA was amplified with primers specific for EBR-pS2, EBR-PR, and EBR-CCND1-(2). *H*, regular MCF-7 cells were treated with hormones as before and ChIPed for ERα and GR followed by amplification with primers specific for EBR-CCND1-(1). All the ChIP experiments were carried out three times, and the data were plotted as relative factor recruitment (averages ± S.E.), setting the number corresponding to ERα recruitment in E2-treated cells as 100.

We noted that majority of the ERα-binding sites tested in this study have AP1-binding sites located within the EBRs (Table 2). AP1 is a dimeric leucine zipper protein mainly composed of either Jun-Jun or Fos-Jun dimers that regulates transcription of genes containing AP1-binding sites also known as 12-*O*-tetradecanoylphorbol-13-acetate DNA response elements (5′-TGA(G/C)TCA-3′) (52). AP1 was shown to be a key regulator of GR activity, and because AP1 directly interacts with GR (53), we hypothesized that GR binding to EBRs occurs via tethering with AP1. To test this possibility, we conducted a ChIP assay using a genetically modified MCF-7 cell line having Tet-off-inducible expression of N-terminally truncated FLAG-tagged c-Jun (Δ2–123, TAM-67) protein. TAM-67 has been demonstrated in a number of studies to be an effective dominant-negative mutant that attenuates AP1 activity in cells (54, 55). MCF-7 (TAM-67) cells grown in the presence or absence of DOX were treated with hormones as described before, followed by ChIP assay. The inducible expression of TAM-67 in cells grown in the absence of DOX was confirmed by Western blotting with an anti-FLAG antibody (Fig. 5*A*). As expected, expression of TAM-67 has no effect on E2-dependent recruitment of ERα to either of the EBRs tested (Fig. 5, *B–D*). By contrast, GR recruitment to EBRs was decreased by 50–60% by the expression of TAM-67 (−DOX) compared with control (+DOX) cells (Fig. 5, *E–G*). To confirm the role of AP1 in GR binding to EBRs, we tested GR loading to an EBR that lacks an AP1-binding site. This EBR is located 2 kb upstream of transcription start site of the *Cyclin D1* gene (Fig. 2*A* and Table 2) and was termed enhancer-1 in the previous study (56). This EBR was also identified in an independent ERα ChIP-chip study conducted by the same group (40). As expected, E2 treatment increased ERα loading to EBR-CCND1-(1), compared with the vehicle control, whereas E2+Dex treatment decreased the ERα recruitment by 24 ± 10% compared with the E2 treatment alone (Fig. 5*H*). Interestingly, there was no recruitment of GR at this EBR in the presence of E2+Dex, in contrast with other three EBRs shown in Fig. 2. These results indicate that WT AP1 binding to its consensus binding sites close to the EREs plays an important role in the recruitment of GR to EBRs. We noted that EBR-CCND1-(1) contains two canonical Sp1-binding sites (Table 2). Previous studies indicate that GR has the ability to interact with Sp1 and regulate Sp1-mediated gene expression (23, 24). Moreover, Sp1 is shown to play an important role in E2-ERα-mediated regulation of all the three genes tested (57–59). However, the lack of GR recruitment to EBR-CCND1-(1) indicated that GR binding to EBR was independent of Sp1 binding. To test this hypothesis, we depleted Sp1 in the MCF-7 cells using siRNA. ChIP analysis showed that Sp1 depletion does not affect GR recruitment to EBRs in the presence of E2+Dex, indicating that Sp1 plays no role in the E2+Dex-assisted loading of GR to EBRs and repression of ERα activity (data not shown).

##### Direct Interaction of GR with ERα Mediates Dex-dependent Recruitment of GR to EBRs

**FIGURE 6.**
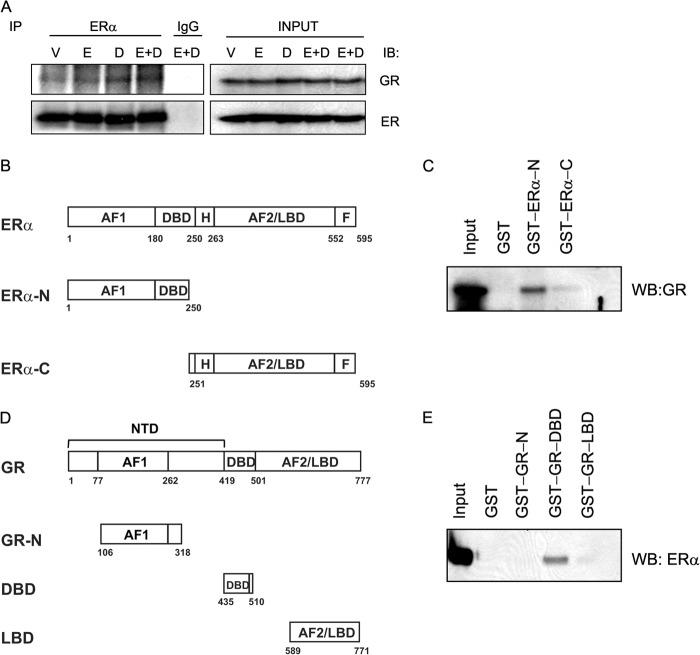
**ERα directly interacts with GR.** MCF-7 cells were grown for 24 h in phenol red-free DMEM supplemented with 10% sFBS and treated with VEH (*V*), E2 (*E*), Dex (*D*), or E2+Dex (*E*+*D*) for 60 min. Next, the cells were harvested and lysed, and the lysate was immunoprecipitated (*IP*) with anti-ERα antibody (*A*) and analyzed for ERα and GR by Western blotting (*WB*). Immunoprecipitations performed with rabbit IgG were used as negative controls, and 5% of the cell lysates (*INPUT*) were assessed for the expression of total ERα and GR by Western blotting. *B*, schematic representation of full-length ERα and N- and C-terminal fragments (ERα-N and ERα-C) of ERα expressed as GST fusion proteins and used in the GST pulldown experiments. *C*, GST pulldown assay demonstrating strong interaction of baculovirus expressed full-length human GR with GST-ERα-N and a weaker interaction with GST-ERα-C and GST. *Input lane* represents 5% of the GR protein used in the pulldown assay. *D*, schematic representation of full-length GR and the GST-tagged GR fragments used in this study. *E*, interaction of *in vitro* translated ERα with GST-GR fusion proteins and GST used as a control. ERα specifically interacts with GST-GR-DBD with little or no interactions with GST-GR-N, GST-GR-LBD, or GST. *Input lanes* represent 5% of ERα protein used in pulldown assays.

The ERα-assisted loading of GR to EBRs also raised the possibility of interaction of these two nuclear receptors in a complex. To test whether GR and ERα interact with each other, we carried out a coimmunoprecipitation study with the whole cell extracts prepared from MCF-7 cells treated with hormones. The endogenous ERα was immunoprecipitated with anti-ERα antibodies, and the immune complex was analyzed for GR and ERα by Western blotting. Our results indicate that endogenous GR interacts with ERα *in vivo*, and this interaction appears to increase when cells are treated with E2+Dex (Fig. 6*A*). To confirm that GR directly interacts with ERα, we carried out *in vitro* GST pulldown assays. We first attempted to express full-length ERα fused with GST protein; however, full-length ERα could not be expressed in *E. coli* because of technical difficulties. We therefore expressed and purified the N-terminal (aa 1–250) and C-terminal (aa 251–595) regions of ERα fused with GST (Fig. 6*B*). The GST-ERα proteins were tested for their ability to interact with baculovirus expressed full-length human GR. We observed that GR directly interacts with ERα with the N-terminal half of ERα, showing stronger interaction with GR than the C-terminal half (Fig. 6*C*), indicating that the AF1 region or ERα-DBD plays a key role in the interaction with GR.

To confirm a direct interaction between ERα and GR and to determine the domain of GR involved in interaction with ERα, a reciprocal GST pulldown assay was carried out. GST fusion proteins encompassing activation function 1 (AF1, termed GR-N, aa 106–318), DBD (aa 435–510), and AF2/ligand binding domain (AF2/LBD, aa 589–771) (Fig. 6*D*) were expressed in *E. coli*, purified and tested for their ability to interact with *in vitro* translated full-length human ERα. We observed that GST-GR-DBD specifically interacts with the *in vitro* translated ERα, whereas GST-GR-LBD and GST-GR-N show no interaction with the protein, clearly indicating that ERα directly interacts with GR through GR-DBD (Fig. 6*E*).

**FIGURE 7.**
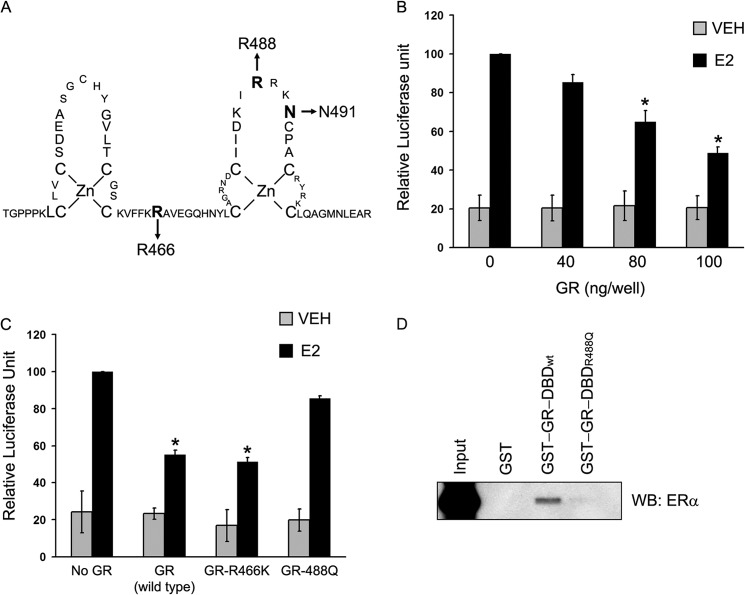
**Arg-488 of the GR-DBD plays an important role in GR-ERα interaction and GR-mediated repression of ERα activity.** *A*, schematic representation of two zinc fingers in the DNA-binding domain of GR. Location of amino acids Arg-466 and Arg-488 and Asn-491 are shown in *bold type. B*, HeLa cells were transfected with expression vectors for ERα (10 ng), GR (0–100 ng), and E2-responsive reporter gene construct, ERE-E1b-Luc (1 μg). The cells were treated with VEH (*gray bars*), 1 nm E2 (*black bars*), and harvested after 24 h for luciferase measurements. The data represent the average luciferase activity ± S.E. of three experiments. Relative luciferase unit corresponding to the E2-treated but no GR-transfected cells was set as 100. The *asterisk* (*) denotes a *p* value of <0.05 in comparison with E2-treated vector transfected group. *C*, HeLa cells were transfected with expression vector for ERα (10 ng), E2-responsive reporter gene construct, and ERE-E1b-Luc (1 μg), along with 100 ng of empty vector or vectors expressing wild type or mutant GRs as shown in the figure. The cells were treated with VEH (*gray bars*) and 1 nm E2 (*black bars*) and harvested after 24 h for luciferase measurements. The data represent the average luciferase activity of three independent experiments. *Error bars*, S.E. The value for relative luciferase unit corresponding to the no GR-transfected E2-treated cells was set as 100. The *asterisks* (*) denote a *p* value of <0.05 in comparison with E2-treated vector transfected group. *D*, GST control, GST-GR-DBD, and GST-GR-DBD_R488Q_ proteins were affinity-purified on glutathione beads and allowed to interact with *in vitro* translated ERα. Bound proteins were resolved by SDS-PAGE and detected by Western blotting with an anti-ERα antibody. *Input lanes* represent 5% of ERα protein used in pulldown assays.

The interaction of GR-DBD with ERα suggested that DNA binding ability of GR could be functionally important for interaction with ERα and repression of ERα activity. The GR-DBD consists of 65 amino acids that fold into two zinc finger domains involved in sequence-specific recognition with the GREs. In an earlier study, 34 point mutants of GR were tested by EMSA for their ability to interact with the consensus GRE. This study showed that 32 of 34 mutants (except R488Q and N491S) had critical roles in recognition with the consensus GRE (33). To determine whether the DNA binding ability of GR plays a role in the repression of ERα activity, we tested wild-type GR and two GR mutants (R466K and R488Q) for their ability to repress ERα activity and interact with ERα (Fig. 7*A*). The mutant R466K is defective in DNA binding, whereas mutant R488Q is similar to WT GR in DNA binding but is defective in tethering cofactors such as Baf60a (60). We transfected ERα-negative HeLa cells with a fixed concentration of ERα expression vector (pCR3.1-ERα), a fixed concentration of vector containing E2-responsive reporter gene (ERE-e1b-luc), and increasing concentrations of GR expression vector (pSG5-GR). The cells were treated with VEH or E2, and luciferase activity was measured. In control cells (with empty plasmid transfected in place of GR), there was a significant increase in luciferase activity in response to E2 treatment compared with VEH control (Fig. 7*B*). Expression of GR repressed the ERα activity in a dose-dependent manner, and a 50% reduction in E2-ERα activity was achieved with 100 ng of GR, compared with cells that received no GR expression vector (Fig. 7*B*). Having established the dose of GR that gives nearly 50% repression of ERα activity, we next conducted a similar ERα transactivation assay with HeLa cells transfected with ERα expression plasmid, ERE-e1b-Luc, along with empty vector, vector expressing wild-type GR, or vector expressing two point mutants (R466K or R488Q) of GR. As expected, wild-type GR showed repressive effect on ERα activity (Fig. 7*C*). A similar level of repression was observed with R466K mutant of GR that does not bind DNA. However, expression of R488Q mutant showed no repressive effect on ERα activity, suggesting that the C-terminal zinc finger domain of the DBD plays an important role in mediating repression of ERα activity. To rule out the possibility that GR-mediated repression of ERα activity was due not to squelching of coregulators but to overexpression of GR in the transactivation assay, we transfected a truncated mutant of GR (GR N556) lacking the AF2/LBD domain that interacts with coregulators (33). GR (N556) repressed E2-ERα activity (data not shown), indicating that TF squelching is unlikely to be the mechanism for GR-mediated repression of ERα activity.

To test whether the loss of repressor function of R488Q mutant GR was due to any effect on ERα-GR interaction, we conducted GST pulldown assays with GST-GR-DBD or GST-GR-DBD (R488Q), expressed and purified in *E. coli*, and *in vitro* translated full-length human ERα. As expected, GR-DBD (WT) showed strong interaction with ERα than the control GST protein, whereas GR-DBD (R488Q) showed no interaction with ERα, suggesting that Arg-488 plays an important role in tethering ERα with GR (Fig. 7*D*). Our data indicate that the C-terminal zinc finger region of GR-DBD plays an important role in the direct interaction of GR to ERα. We suggest that this interaction leads to GR-mediated repression of ERα transcriptional activity.

## DISCUSSION

GCs have been shown to inhibit ERα activity (39), E2-ERα target gene expression (9, 39), and ER-positive breast cancer cell proliferation *in vitro* (9, 61) and in the mouse xenograft model (38). However, the molecular mechanisms underlying such inhibitory effects are poorly understood. We show that Dex inhibits E2-induced MCF-7 cell proliferation and expression of ERα target genes *pS2* and *Cyclin D1*. This effect is mediated by loading of GR to EBRs in response to E2+Dex treatment. The inhibition of ERα activity by GR requires direct protein-protein interaction of ERα with GR through its DNA-binding domain. Mutational studies suggest that Arg-488 located in the C-terminal zinc finger domain of GR plays an important role in this interaction, underscoring the importance of C-terminal zinc finger interface in the GR-ERα recognition and GR-mediated interference in ERα activity.

A previous study showed that ligand-bound GR transcriptionally activates estrogen sulfotransferase, a key enzyme that deactivates E2 by sulfonation (38), suggesting an indirect mechanism for GR-mediated interference of ERα activity. However, we demonstrate co-occupancy of GR and ERα, which decreases loading of ERα and its coactivator SRC-3 to EBRs, indicating that more than one mechanism may be involved in GR regulation of ERα activity. Both ERα and GR regulate gene expression by directly interacting with their respective response elements (EREs or GREs) or by indirectly tethering to other TFs such as AP1 and NFκB. Because binding of GR and ERα to their cognate DNA response elements is highly specific, it is unlikely that GR and ERα form a heterodimer at the ERE (62). Thus, it is fair to speculate that GR is recruited to EBRs via protein-protein interactions as opposed to protein-DNA interaction between GR and EREs. In this regard, we note that none of the three EBRs tested in this study harbors GREs but contains AP1 or Sp1-binding sites. Our data indicate that the presence of an AP1 site in the EBRs and interaction of GR with AP1 is a prerequisite for GR loading to EBRs. Because Dex alone was unable to induce GR recruitment to either of these sites and Dex+E2 led to a robust recruitment of GR, the results indicate an ERα-coordinated recruitment of GR to these EBRs that does not contain GREs. Similarly, depletion of Sp1 and subsequent ChIP assays show that recruitment of GR to EBRs is independent of Sp1 binding.

We note that Dex-mediated repression of E2-ERα activity and inhibition of E2-mediated cell proliferation are partial. Higher concentration of Dex (up to 10 μm) does not lead to complete inhibition of ERα activity, indicating that partial effects are not due to subsaturated levels of hormone-bound GR in the cells. Although a molecular mechanism of GR/AP1-mediated displacement of ERα is not clear, we suggest that AP1 stabilizes GR occupancy on the EBRs through protein-protein interactions in the absence of canonical GREs and therefore levels of expression of both AP1 and GR and the accessibility of AP1-binding sites in the EBRs could determine the overall transcriptional outcome of ERα target genes. We observe that recruitment of GR to EBRs is not a favorable event. There is only 10-fold enrichment (over the vehicle-treated group) of GR to *pS2*, *PR*, and *CCND1-(2*) EBRs, compared with >200-fold enrichment of GR observed at the FKBP5 GBR that contains a canonical GRE (Fig. 2, *E–G*, *versus* Fig. 4*F*). Therefore, partial Dex-mediated interference in ERα activity is not surprising. Moreover, nuclear receptors and their coregulators dynamically interact with regulatory sites in chromatin (63) where stoichiometric ratios of the partner proteins such as GR/AP1 and other epigenetic gene regulatory mechanisms could dictate the overall transcriptional outcome of individual ERα target genes.

Our finding that the DNA-binding domain of GR directly interacts with ERα raises the possibility of AP1-GR-mediated global interference in ERα activity. Most point mutations within the two zinc finger domains of GR-DBD affect sequence-specific DNA binding property of GR. However, certain mutations such as arginine 466 to lysine (R466K) or arginine 488 to glutamine (R488Q) attenuate hormone-dependent transactivation potential of GR. The mutant R466K is defective in DNA binding, whereas R488Q displays DNA binding properties similar to WT GR. Our data show that Arg-488 (but not R466) is required for regulation of GR-mediated interference in ERα function. The fact that GR-R488Q binds to DNA but not to ERα implies that repression of ERα activity by GR mainly occurs through protein-protein interactions. Previous studies demonstrated that the Arg-488 residue of GR-DBD domain is critical for direct interaction with BRG1-associated factor 60a (Baf60a). Baf60a is a component of the ATP-dependent chromatin remodeling complex (SWI-SNF) that remodels chromatin structure and creates DNase I-hypersensitive sites (short regions of DNA that are highly sensitive to cleavage by DNase I), at the GR-binding regions (60). Thus, transcriptional inactivity of GR-R488Q could be attributed to the impairment of its interaction with the SWI-SNF complex. WT GR physically associates with NFκB and AP1 and represses their transcriptional activity (21, 64). A previous study showed that GR-R488Q, although it physically interacts with NFκB, is unable to repress NFκB-stimulated transcriptional activity, whereas the repression of AP1 activity remains unaffected by this mutant (65, 66). Furthermore, global gene expression profiling conducted on human embryonic kidney 293 cells identified differentially regulated genes by WT-GR and GR-R488Q mutant (67). Most genes affected by the R488Q mutation seem to be involved in the control of transcription and cell growth, further suggesting a key role of the C-terminal zinc finger interface in affecting selectivity in gene regulation (67). We note that in our case, R488Q mutation affects direct interaction with ERα as well as its transcriptional activity. We therefore suggest that ERα and NFκB may share similar but distinct mechanisms of inhibitory cross-talk with GR.

**FIGURE 8.**
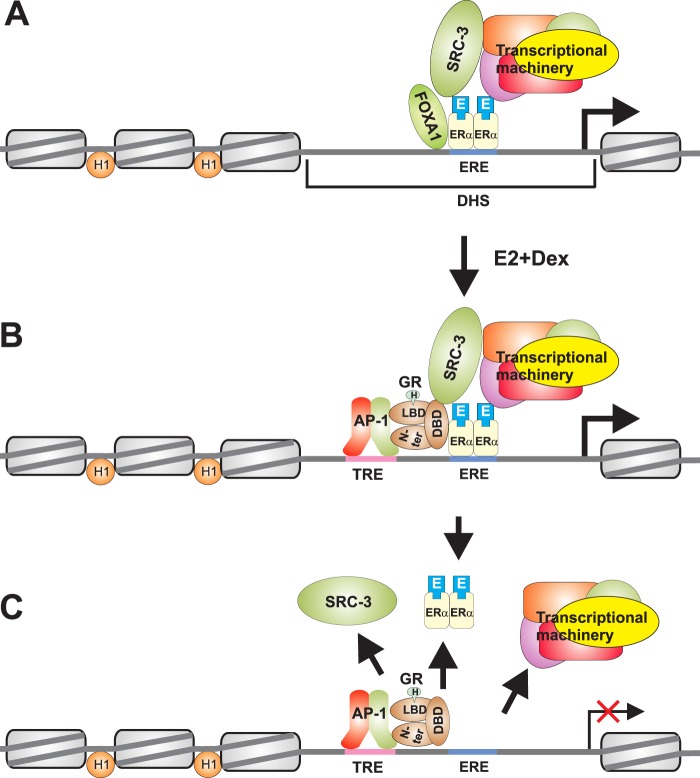
**A model representing a putative mechanism of GR-mediated repression of ERα activity.** *A*, FOXA1 and other chromatin remodeling enzymes create accessible chromatin regions as reflected by the DNase I hypersensitivity in the EBRs and facilitate the recruitment of E2-bound ERα, its coactivator SRC-3, and other chromatin-modifying activities. The ERα-SRC-3 complex in turn interacts with the components of general transcription machinery increasing the rate of transcription. *B*, E2+Dex treatment facilitates AP1 binding to 12-*O*-tetradecanoylphorbol-13-acetate response elements (*TRE*) located in the EBRs; AP1 tethers GR to the EBRs. *C*, tethered GR interacts with ERα and destabilizes ERα-SRC-3 complex reducing the rate of ERα-mediated transcription.

ERα binding to target sites in chromatin occurs through a variety of mechanisms, including direct binding to consensus EREs, indirect recruitment via other TFs, or binding through composite response elements where receptor and other TFs bind next to each other and regulate transcription (68). ERα-binding sites are generally present upstream of the ERα-regulated genes, with relatively few sites located downstream of the protein coding regions. Moreover, recent genome-wide studies showed that most ERα-binding sites are located far from the transcriptional start sites of their target genes (40, 69, 70). These studies demonstrated the pleiotropic nature of ERα, because agonist-bound receptor could both activate (∼30%) and repress (∼70%) different sets of target genes (34). A key question therefore arises: Does GR preferentially associate with certain EBRs, and does sequence composition of the binding region play any role in the coassociation of ERα and GR? It would also be interesting to see whether GR recruitment to EBRs affects both ERα up- and down-regulated genes. Thus, a genome-wide ChIP-Seq is warranted to address the binding preference of GR to ERα binding loci, and that compared with global expression profiling would determine the functional outcome of GR and ERα interaction in the context of chromatin. Our studies show that liganded ERα is required for tethering GR to ERα. We propose that ERα in response to hormone stimulation recruits FOXA1 or other chromatin remodeling activities to remodel the chromatin structure at the target EBRs as reflected by the presence of inducible DNase I-hypersensitive sites at EBRs (71) (Fig. 8*A*). The increased chromatin accessibility at EBRs exposes the AP1-binding sites, which allow tethering of GR to EBRs (Fig. 8*B*). The AP1-tethered GR interacts with ERα through it C-terminal zinc finger region (Arg-488) and destabilizes the ERα-SRC-3 complex leading to the repression of ERα activity (Fig. 8*C*). Alternatively, the ERα and GR complex is formed in the nucleoplasm and is brought to the EBRs in the presence of hormones where it is tethered to the prebound AP1. In either case, the opening of higher order chromatin structure following E2-ERα recruitment, exposure of AP1-binding sites, and subsequent interaction of GR with AP1 play a key role in orchestrating GR loading to EBRs and repression of ERα activity. Our model is supported by the observation that ERE-CCND1-(1), which lacks an AP1-binding site, fails to show E2+Dex-mediated GR recruitment to EBR.

In conclusion, we demonstrate a unique mechanism that GCs employ to regulate ERα activity and ER-positive breast cancer cell proliferation and survival. The role of GR in breast cancer is complex, and there are limited and contradictory data suggesting a protective or prognostic value of GR in breast cancer treatment. Studies show an inverse correlation between the expression levels of GR and ERα in several breast cancer cell lines (72). Because ER-positive breast cancer cells rely mostly on ERα signaling pathway for their proliferation, and activation of GR has negative effect on it, it is conceivable that GR expression and activation is associated with better treatment outcome in ER-positive breast cancers. Our findings suggest that activation of ERα by E2 is important for GR to regulate ERα activity and ERα-mediated cell growth. This raises the possibility that strategies targeting the GR signaling pathway in breast cancer could be particularly beneficial for ERα-positive patients. Our study provides a framework to understand the molecular mechanism underlying differential response of breast tumors to GCs and establish a foundation for pursuing GC treatment to enhance the safety and effectiveness of endocrine therapy for breast cancer patients.

## Footnotes

2 The abbreviations used are:

E2: 17-β-estradiol
ER: estrogen receptor
GR: glucocorticoid receptor
NFκB: nuclear factor κ-light-chain-enhancer of activated B-cells
FOXA1: forkhead box protein A1
AP1: activator protein 1
Sp1: specificity protein 1
ERE: estrogen response element
EBR: ERα-binding region
GBR: GR binding region
Dex: dexamethasone
DOX: doxycyclin
GC: glucocorticoid
SRC: steroid receptor coactivator
DBD: DNA-binding domain
TF: transcription factor
VEH: vehicle
aa: amino acid(s)
sFBS: hormone-depleted, charcoal-stripped FBS
qPCR: quantitative polymerase chain reaction.
